# A Public-Private Partnership Model for Obesity Prevention

**Published:** 2009-06-15

**Authors:** Terry T Huang, Amy L. Yaroch

**Affiliations:** Eunice Kennedy Shriver National Institute of Child Health and Human Development, Bethesda, Maryland; The Center for Human Nutrition, Omaha, Nebraska

## To the Editor:

In the January 2009 issue of *Preventing Chronic Disease*, McDermott et al (1) and Harris et al (2) offer a glimpse of the potential to work across sectors to generate solutions for the obesity epidemic. Obesity has persisted in the United States and is increasing worldwide despite years of research to combat it. In this issue of  *Preventing Chronic Disease*, we have proposed, through a series of articles, a systems-oriented, multilevel framework to realign strategies and resources to address this public health problem. A key feature of this framework is building capacity across the public and private sectors to mobilize a coordinated effort to change the environment that constrains healthy behaviors, such as healthy diet and physical activity, and enables unhealthy ones. We argue that public health must collaborate not only with government organizations, community coalitions, academia, and mass media but also the corporations that control and shape our food system.

Public health can benefit from industry resources for research and industry expertise in such areas as food formulation and marketing, just as industry can benefit greatly from public health's ability to design effective programs for health promotion or to develop health-conscious business models.

Past negative experiences, for example, with tobacco companies, have left many academics and public health professionals wary of engaging the food industry. Profit motives of corporations are assumed to be inconsistent with public health goals (3), but tobacco use is not required for survival and the same cannot be said about food. In fact, the food system is much more complex, and to a great extent, the accessibility, availability, and marketing of foods are shaped by the companies that make up the global food chain (4). Public health cannot win a war with the food industry, because the same companies that produce unhealthy foods also produce healthy ones. Therefore, the question for public health is not to treat the food industry as the enemy but to capitalize on the industry's need for a positive image and long-term business viability.

Like any other relationship, public-private partnerships must be built on trust, and trust requires a mutual commitment to open and honest dialogue over time. Both common ground and barriers to collaboration must be discussed. Once an agreement is reached to formally establish a public-private partnership, transparency, accountability, a sound governance structure, and well-defined leadership are keys to partnership success (5).

The need for broader public-private partnerships does not mean that such partnerships are not entered into cautiously or that government legislation to change the food environment is not needed. In fact, what food companies worry most about is that voluntary changes to their products or marketing strategies will make them less competitive in the market. This is why industry is slow to adopt voluntary efforts and such efforts are generally ineffective. Public-private partnerships can expedite the adoption of new rules that apply to a wide range of competing companies. The right public-private partnership can engender cooperation rather than hostility from corporate partners.

The government can foster public-private partnerships. Government agencies can convene the right participants and maintain the interests of both corporations and public health. In addition, private resources for research can be filtered through existing legal frameworks in the government so that private and public funding are mixed and diffused to a broad range of projects. As such, public-private partnerships mediated by a government entity can help shield researchers from the liability of bias that may come with using private dollars. The government can also monitor research to ensure its objectivity.

In a societal approach to combating obesity, every participant, including the food industry, has a role. Public-private partnerships can enhance rather than hinder the development of effective and sustainable solutions.
